# Indoors illumination and seasonal changes in mood and behavior are associated with the health-related quality of life

**DOI:** 10.1186/1477-7525-6-56

**Published:** 2008-08-01

**Authors:** Sharon Grimaldi, Timo Partonen, Samuli I Saarni, Arpo Aromaa, Jouko Lönnqvist

**Affiliations:** 1 National Public Health Institute, Department of Mental Health and Alcohol Research, Helsinki, Finland

## Abstract

**Objective:**

Seasonal changes in mood and behavior are common in a general population, being of relevance to public health. We wanted to analyze whether the HRQoL is associated with the seasonal changes in mood and behavior. Because the shortage of exposure to daylight or artificial bright light has been linked to the occurrence of the seasonal changes, we wanted to know whether illumination indoors contributes to the HRQoL.

**Methods:**

Of the sample of 7979 individuals, being representative of the Finnish general population aged 30 and over, 88% were interviewed face to face, and 84% participated in the health status examination after which the self-report assessment of the HRQoL and the seasonal changes in mood and behavior took place. The illumination levels experienced indoors were asked during the interview and the 12-item General Health Questionnaire (GHQ-12) was filled in before the health examination.

**Results:**

The HRQoL was influenced by both the seasonal changes in mood and behavior (P < 0.001) and the illumination experienced indoors (P < 0.001). Greater seasonal changes (P < 0.001) and poor illumination indoors (P = 0.0035) were associated with more severe mental ill-being.

**Conclusion:**

The routinely emerging seasonal changes in mood and behavior are associated with the HRQoL and mental well-being. Better illumination indoors might alleviate the season-bound symptoms and thereby enhance the HRQoL and mental well-being.

## Introduction

Exposures to light, or the light-dark transitions, are needed for reset of the principal circadian clock on a daily basis. The principal circadian clock, which is located in the suprachiasmatic nuclei of the anterior hypothalamus in the brain, also reacts to changes in the length of day [1] and thereby tunes the drive to its targets [2]. Changes of season challenge these time-keeping mechanisms of action as, for instance, the evening-active cells yield the dominance to the morning-active cells within the principal circadian clock following the shortening of the length of day and the shortage of daylight in the fall [3]. Individuals with recurrent major depressive episodes in a particular period of the year have seasonal affective disorder [4]. Patients with these seasonal symptoms have impairment in the quality of life (QoL) during winter but improve with light therapy or antidepressants [5]. Mental functioning in particular tends to be compromised and thereby lowers the health-related quality of life (HRQoL) as compared to the general population. Interest in the assessment and significance of the HRQoL has increased in recent years [6].

The shortening length of the day tends to affect mental well-being [7] and to trigger the occurrence of season-bound symptoms at the population level [8]. The natural daylight is considered to improve mental well-being, or the feeling of general well-being, whereas artificial light exposures may be beneficial as well [9].

### Aims

Our aim was to study the associations of HRQoL with exposure to illumination and with seasonal changes in mood and behavior. To be specific, we aimed at elucidation of associations, if any, of the 15D Health-Related Quality of Life Instrument (15D) sum and item scores with the global and six item scores on the Seasonal Pattern Assessment Questionnaire (SPAQ). Because the shortage of exposure to daylight or artificial bright light has been linked to the occurrence of the seasonal changes, we wanted to know whether illumination indoors contributes to the HRQoL. Moreover, because mental health is a major part of the HRQoL, we analyzed the 12-item General Health Questionnaire (GHQ-12) sum and item scores in addition to the 15D which contains two items on depression and distress only.

## Methods

The data for this study was obtained from a national health examination survey. The study (Health 2000) was carried out in Finland, a north-eastern European country with about 5 million inhabitants. The fieldwork with data collection was carried out between September 2000 and July 2001. The two-stage stratified cluster sampling design was planned by Statistics Finland. The sampling frame comprised adults aged 30 years and over living in mainland Finland. This frame was regionally stratified according to the five university hospital regions, each containing roughly one million inhabitants. From each university hospital region or catchment area, 16 health care districts were sampled as clusters (80 health care districts in the whole country, including 160 municipalities). The 15 biggest health care districts in the country were all selected in the sample and their sample sizes were proportional to population size. The remaining 65 health care districts were selected by systematic probability proportional to size sampling in each stratum, and their sample sizes (ranging from 50 to 100) were equal within each university hospital region, the total number of persons drawn from a university hospital region being proportional to the corresponding population size. The 80 health care districts were the primary sampling units, and the ultimate sampling units were persons who were selected by systematic sampling from the health centre districts. From these 80 health care districts, a random sample of individuals was drawn using the data provided by Population Register Centre. Its population information system contains the official information for the whole country on the Finnish citizens and aliens residing permanently in Finland.

For this study, all the persons aged 30 or over (n = 8028) identified and selected by The Social Insurance Institution of Finland were contacted. Interviewers attended training sessions on the specific themes that were to be covered in the computer assisted interviews. During the interviews, the respondents were handed an information leaflet, an informed consent form for signing, and a questionnaire containing self-reports such as the SPAQ, the 15D, the GHQ-12 and the Beck Depression Inventory (BDI) that interviewees were asked to fill in and bring along to the health status examination.

Of the final sample of 7979 persons, 6986 (88%) were interviewed at home or institution face to face and 6354 (80%) attended the health status examination in a local health center or equal setting, while 416 took part in the health status examination at home or in an institution. Overall, 84% participated either in the health status examination proper or in the examination at home. All the methods are reported more in detail on the Internet site of the Health 2000 (for details, please see ).

### Health-related quality of life

The HRQoL was measured using two instruments, the 15D and the GHQ-12. The 15D instrument measures 15 dimensions including mobility, vision, hearing, breathing, sleeping, eating, speech, elimination, usual activities, mental function, discomfort and symptoms, depression, distress, vitality, and sexual activity [6]. It contains five ordinal levels on each dimension, and the respondent is instructed to choose from each item the level which best describes the current health status. 15D is a generic, comprehensive, standardized measure which yields both a profile and a single index score. Higher scores indicate better levels of the HRQoL. The index of zero to one, representing the overall HRQoL, is calculated by using a set of population-based preference or utility weights. The 15D scores are highly reliable and can be generalized in Western-type societies (for further information, please see ).

In addition to the 15D and its depression and distress dimensions, we wanted to assess more in detail the part of the HRQoL to which mental well-being contributes by using the 12-item GHQ. It is scored on a four-point Likert-like scale (less than usual, no more than usual, rather more than usual, or much more than usual), yielding a sum score ranging from 0 to 36. Higher scores indicate greater mental ill-being. The GHQ was developed in the 1970s with the purpose to evaluate mental health and has been applied in a range of settings and cultures [10]. Its original version contains 60 items, but the instrument is available as shortened forms, like as the GHQ-12. This version evaluates whether the individual complains about a recent symptom or behavior. The GHQ-12 is documented well, easy to complete and valid as a screening tool [11]. It is a valid measure of the psychological symptoms at population level, especially in the areas of anxiety and depression [12]. According to the analysis of data derived from the Health 2000 Study, the threshold value of 4 was taken to indicate ill health (the scores of 0 to 4 assigned as low and those of 5 to 36 as high).

### Seasonal changes in mood and behavior

Seasonal changes in mood and behavior were measured using items taken and adapted from the SPAQ [13]. Two modifications were made to the original scoring as follows. Each item was scored from 0 to 3 (none, slight, moderate or marked change), not from 0 to 4 (none, slight, moderate, marked or extremely marked change), with the sum or global seasonality score (GSS) ranging from 0 to 18. Higher scores indicate greater seasonal changes. In addition, the SPAQ has a question: "If you experience changes with the seasons, do you feel that these are a problem for you?". This item was scored from 0 to 4 (none, mild, moderate, marked or severe problem), not from 0 to 5 (none, mild, moderate, marked, severe or disabling problem). The questionnaire was translated into Finnish and then back-translated in order to revise the linguistic accuracy. Since the seasonal changes in mood and behavior were assessed with a modified questionnaire, we tested earlier its psychometric properties and found them to be good in the adult population of ours [14], yielding a population-based distribution of the GSS across individuals similar to the original one [15]. The modified questionnaire was thereafter applied for assessment using the cut-point of 7 (the scores of 0 to 7 assigned as low and those of 8 to 18 as high) which is similar to the original case-finding criteria [15].

### Experienced exposure to illumination

Exposure to illumination was measured using two items which had not been validated earlier. Concerning the experienced indoors illumination, two items of the experienced lighting levels were analyzed. Poor lighting at home (yes or no) and insufficient lighting at work (not present or no problem, troubles to some extent, troubles quite a lot, or troubles exceedingly) were assessed as part of in the computer assisted interview. The sum of the scores on the two items was calculated and categorized for the analysis. Higher scores indicate poorer lighting conditions.

### Other self-reports

We decided that it was important to include a measurement of depression as an explanatory variable in the analysis. Therefore, we assessed the behavioral manifestation and symptom intensity of depression using a modification of the 21-item BDI [16] as adapted and validated for the Finnish population (for further information, see ), with a sum score ranging from 0 to 55. The modified questionnaire was thereafter applied for the case-finding definition using the cut-point of 9 (the scores of 0 to 9 assigned as low and those of 10 to 55 as high). Higher scores indicate more severe depressive symptoms. However, no diagnosis of depressive disorder can be assessed with the BDI.

Other variables used in the analysis of data were as follows. As part of the assessment, the participants filled in items concerning their leisure time exercise, alcohol use during the past 12 months, activities outdoors, and social activities. The intensity of physical exercise was categorized as follows: low (no strenuous exercise such as reading, watching television or handicraft), medium (lightly strenuous exercise such as walking or bicycling for four or more times a week), keep-fit (fitness training for three or more hours a week), and sport (sports for several times a week). The frequency of alcohol use was categorized as follows: none, low (once to six times a year), medium (once to four times a month), and high (twice to seven times a week). The frequencies of social activities (meeting relatives, friends or neighbors) and of activities spent outdoors (exercise, hunting, fishing, gardening or other outdoor recreation) were categorized as follows: low (less than once a year), medium (once a year to twice a month), and high (once to seven times a week).

### Ethics

The National Public Health Institute coordinated and implemented the study project in collaboration with the Ministry of Social Affairs and Health. It provided a written informed consent to each participant, giving a full description of the protocol before signing it. The procedures were according to the ethical standards of the responsible committee on human experimentation and with the Declaration of Helsinki, its amendments and revision.

### Statistics

The data were weighted to take into account the sampling design and to reduce the bias due to non-response. The R project for Statistical Computing (R, version 2.2.1) was applied for, and its survey Package, available through the Comprehensive R Archive Network family of internet sites , was run for analysis of the stratified data using survey-weighted generalized linear models.

We wanted to know whether there was any association of the health-related quality of life with the seasonal changes in mood and behavior or with the illumination levels. To that end, first, a multivariate regression model using the indexed sum score on the 15D and another model using the categorized sum score on the GHQ-12 as the dependent variable were computed. For both models, the following explanatory variables: the sex, age in four categories (30 to 45, 46 to 60, 61 to 75 or 76 to 99 years), education in three categories (low, middle or high), marital status in two categories (living alone or with someone), area of living in two categories (the southern or northern part of Finland), physical exercise, alcohol use, the GSS in two categories (0 to 7 or 8 to 18), illumination levels experienced in two categories (not poor and not a problem, or poor or of trouble to any extent), activities outdoors, and social activities. In the former model, the BDI sum score in two categories (0 to 9 or 10 to 55) were in addition included as a covariate. Second, the two models in which the GSS was replaced by the six items of which the GSS is comprised were computed in order to elucidate which of the seasonal changes explained the association best.

## Results

To see whether mental health contributes to the HRQoL in the current sample, we computed two univariate regression models. The explanatory variable was the 15D item score on depression in the one and the 15D item score on distress in the other, whereas the 15D sum score was the dependent variable in both models. Both 15D items contributed to the HRQoL significantly and equally (the adjusted R^2 ^of 0.30 for both).

### Determinants of the health-related quality of life

First, we found that both the seasonal changes in mood and behavior and the experienced illumination indoors contributed independently to the HRQoL, since both the GSS (t = -13.34, P < 0.001) and the illumination score (t = -4.75, P < 0.001) were significantly associated with the 15D sum score in the two univariate regression models.

Second, we confirmed that both the seasonal changes in mood and behavior and the experienced illumination indoors contributed independently to the HRQoL, since both the GSS (t = -8.70, P < 0.001) and the illumination score (t = -4.10, P < 0.001) were significantly associated with the 15D sum score still after controlling for known and potential confounding factors in the multivariate regression model (Table 1). This finding was supported by the post-hoc test comparisons of the GSS and its six items between the two groups categorized by the experienced illumination indoors, which showed no significant association.

**Table 1:** Regression analysis of the determinants of the sum score on the 15D.

**Variable**	**Estimate**	**Standard error**	**t value**	**P value**
Female sex	-0.00054	0.0019	-0.29	0.77
Aged 45 to 60	-0.014	0.0018	-7.68	<0.0001
Aged 61 to 75	-0.030	0.0069	-4.31	<0.0001
Aged 75 to 99	-0.12	0.078	-1.50	0.13
Medium education	0.0034	0.0027	1.24	0.21
High education	0.0036	0.0030	1.22	0.22
Living together	0.0025	0.0022	1.12	0.26
Location in the north	-0.0017	0.0028	-0.77	0.44
Medium exercise	0.0067	0.0026	2.54	0.011
Fitness exercise	0.011	0.0027	4.08	<0.0001
Sport exercise	0.0094	0.0051	1.84	0.066
Medium alcohol intake	0.0048	0.0023	2.14	0.033
High alcohol intake	0.0026	0.0026	1.010	0.31
No alcohol intake	-0.0023	0.0038	-0.59	0.56
High GSS	-0.021	0.0024	-8.70	<0.0001
High BDI	-0.068	0.0029	-23.63	<0.0001
Low illuminance levels	-0.011	0.0026	-4.10	<0.0001
Medium outdoor activities	-0.0030	0.0028	-1.06	0.29
High outdoor activities	0.0016	0.0029	0.55	0.58
Medium social activities	0.0013	0.0024	0.53	0.60
High social activities	0.0030	0.0025	1.17	0.24

In the subsequent multivariate regression model, we analyzed which seasonal changes in mood and behavior were of significance. We discovered that the seasonal changes in energy level (t = -4.26, P < 0.001), mood (t = -3.62, P = 0.00031) and social activity (t = -2.18, P = 0.029) were the significant explanatory variables.

### Determinants of mental well-being

To start with, we found that both the seasonal changes in mood and behavior and the experienced illumination indoors contributed independently to mental well-being, since both the GSS (t = 12.63, P < 0.001) and the illumination score (t = 2.92, P = 0.0035) were significantly associated with the GHQ-12 sum score in the two univariate regression models.

Thereafter, we confirmed that both the seasonal changes in mood and behavior and the experienced illumination indoors contributed independently to mental well-being, since both the GSS (t = 8.94, P < 0.001, odds ratio of 2.97, 95% confidence interval of 2.34 to 3.76) and the illumination score (t = 2.37, P = 0.018, odds ratio of 1.39, 95% confidence interval of 1.06 to 1.82) were significantly associated with the GHQ-12 sum score after controlling for known and potential confounding factors in the multivariate regression model (Table 2).

**Table 2:** Regression analysis of the determinants of the sum score on the 12-item General Health Questionnaire.

**Variable**	**Estimate**	**Standard error**	**t value**	**P value**
Female sex	0.31	0.12	2.56	0.010
Aged 45 to 60	-0.088	0.11	-0.82	0.41
Aged 61 to 75	-0.64	0.44	-1.46	0.14
Aged 75 to 99	1.28	0.99	1.30	0.20
Medium education	-0.00047	0.16	-0.0030	1.00
High education	0.11	0.16	0.70	0.49
Living together	-0.29	0.11	-2.51	0.012
Location in the north	-0.098	0.13	-0.74	0.46
Medium exercise	-0.16	0.13	-1.19	0.23
Fitness exercise	-0.10	0.16	-0.62	0.54
Sport exercise	-0.56	0.51	-1.09	0.28
Medium alcohol intake	0.091	0.13	0.69	0.49
High alcohol intake	0.19	0.17	1.14	0.25
No alcohol intake	0.45	0.21	2.22	0.027
High GSS	1.09	0.12	8.94	<0.0001
Low illuminance levels	0.33	0.14	2.37	0.018
Medium outdoor activities	-0.36	0.15	-2.45	0.014
High outdoor activities	-0.40	0.15	-2.62	0.0089
Medium social activities	-0.24	0.13	-1.86	0.064
High social activities	-0.49	0.14	-3.46	0.00057

Finally, we analyzed which seasonal changes in mood and behavior were of significance in the subsequent multivariate regression model. We discovered that the seasonal changes in mood (t = 2.77, P = 0.0057), appetite (t = 2.54, P = 0.011), social activity (t = 2.21, P = 0.027) and energy level (t = 2.11, P = 0.035) were the significant explanatory variables.

## Discussion

Herein, we wanted to analyze whether the HRQoL is associated with the seasonal changes in mood and behavior. Because the shortage of exposure to daylight or artificial bright light has been linked to the occurrence of these seasonal changes, we wanted to know whether illumination indoors contributes to the HRQoL. Not only the seasonal changes in mood and behavior, but also poor illumination levels at home or at a working place may therefore have a negative effect on the QoL in general, the HRQoL in particular and mental well-being in specific.

Our results demonstrate that the HRQoL is influenced by both the illumination experienced indoors and the seasonal changes in mood and behavior. Concerning the HRQoL the negative effect of poor illumination indoors equals to the positive effect gained with regular physical exercise having the intensity of fitness training. The intensity of seasonal changes in mood and behavior has a negative effect on the HRQoL that was second to the intensity of depressive symptoms only and greater than that of age for instance. Of the seasonal changes in mood and behavior, those in energy level, mood and social activity were of significance to the HRQoL.

Greater social activities, more activities outdoors and living together were positively associated with better mental well-being. On the other hand, greater seasonal changes and poor illumination indoors are significant factors which were associated with worse mental ill-being. The intensity of seasonal changes in mood and behavior has a negative effect on mental well-being that was second to none. In other words, its effect was greater than that of the sex, age, education, outdoor or social activities for example, and the degree of these seasonal changes similar to that of winter blues yields the odds ratio of 2.97 for suffering from mental ill-being to a marked extent. Of the seasonal changes in mood and behavior, those in mood, appetite, social activity and energy level were of significance to mental well-being. Here, the negative effect of poor illumination indoors is greater than the positive effect gained with regular physical exercise having the intensity of sports activities, and bears the odds ratio of 1.39 for suffering from mental ill-being to a marked extent.

Seasonal changes in mood and behavior are common in a general population, thereby being of relevance to public health. Illumination levels indoors may be enhanced with the architectural and design solutions, and the season-bound changes in mood and behavior can be alleviated or even prevented with the use of scheduled light exposures [17]. These practices may converge, and both risk factors may be alleviated with innovations taking advantage of light exposure schedules.

Individuals having seasonal affective disorder have a compromised QoL during winter against which scheduled light exposures provide alleviation [4]. If a major depressive episode is present, the QoL may be decreased further. Patients with seasonal affective disorder usually have an adequate level of physical activities but suffer from poor mental functioning in particular when depressed [18]. When summer comes, the season-bound symptoms disappear and the QoL on average and mental health, health perceptions and social functioning in specific improve [19].

Our findings herein suggest that light exposure and illumination levels are important to the QoL, the HRQoL and mental well-being. Bright light exposure indoors can increase the level of vitality, quality of sleep, physical activity, energy level and social activities, while it decreases the intensity of depressive symptoms even in persons having no seasonal changes in mood or behavior [9]. The HRQoL and distress appear to improve with bright light as well. In addition to light exposure, physical exercise enhances the QoL and mental well-being. Fitness training decreases depressive symptoms [20], whereas bright light decreases the intensity of season-bound symptoms such as increased appetite, carbohydrate craving and prolonged sleep as compared with physical exercise alone [21]. Melatonin is a third treatment option that has been studied earlier in individuals having the seasonal changes in mood and behavior, and it improves the health-related quality of life, the quality of sleep, and mood [22]. To sum up, the scheduled interventions which give feedback to the principal circadian clock during appropriate periods of the day have the potential to be of benefit to not only conditions due to the circadian rhythm disturbances in specific [23] but also the HRQoL and mental well-being in general.

Not only the experienced levels of illumination indoors but also the perception of environment plays an important role in the QoL. Our results herein support this view and demonstrate that greater social activities, more activities outdoors and living together have a positive association with better mental well-being. The increased long-term stress response is associated with the perceptions of instability and decreased control as well as a lack of social support [24]. It may explain why some disadvantaged populations experience higher morbidity and mortality rates for instance. Non-medical determinants of health, however, affect people differently during different periods in life. Changes in the physical and social activities, such as those that affect income and financial security, social circles, leisure, physical and mental health and abilities, are known to be linked to distress but occur to one during different schedules. Therefore, even if there were no physiological pathway from the habitat to the health status and HRQoL, barriers to the physical and social activities are likely to have an impact on an individual basis.

### Strengths and limitations

Our data were collected as part of a big nationwide sample which was assessed with a personal interview face to face, a comprehensive health examination protocol and a questionnaire delivery. Our findings are representative of the general population aged over 30 living in Finland, a northern European country, and can therefore be generalized to concern any population with a similar standard of living at the time of the study. Seasonal changes in mood and behavior are a common phenomenon, but the prevalence rates appear to vary between countries and some populations who have lived for longer at high northern latitudes may have adapted better than others [25]. Milder forms of seasonal affective disorder are more prevalent in more northern latitudes, whereas the prevalence of affective disorder with the seasonal pattern is equal between southern and northern parts of Europe for example [26]. Variations in the key circadian clock genes and in their regulation through the feedback the principal circadian clock receives may make a difference [27].

Our limitation was the cross-sectional study design, and therefore we cannot present any causal deduction concerning the associations we found. Another limitation was the use of self-reports of the seasonal changes in mood and behavior, and of the illumination levels. However, the former questionnaire is retrospective to the routine seasonal changes during lifetime, and it has high internal consistency [28] and two-month test-retest reliability [29]. The latter self-report is a subjective estimate of the indoor lighting conditions only.

### Research implications

Our findings herein suggest that illumination levels indoors are of importance to mental well-being. They may therefore stimulate further research aiming at designing optimal working and living environments in terms of lighting conditions. Current indoor lighting standards are based on specifications concerning the visual requirements. If the non-visual effects of light exposure to the eyes which contribute to the seasonal changes in mood and behavior were to be considered, novel codes and standards that influence the choice of lighting technologies and the design of indoor environments could be developed and implemented to be in use.

### Clinical implications

Such solutions concerning the use of indoor lighting applications will be of clear benefit to those 1,226,531 persons in approximate, which equals 39% of the whole population aged 30 and over living in Finland, who routinely suffer from the seasonal changes that emerge during winter and lead to winter blues. They may also be of benefit to patient populations such as those with seasonal affective disorder, or bipolar or recurrent major depressive disorders with a seasonal pattern, in particular. In addition, our findings herein and subsequent research activities on the design of indoor environments may bear relevance to the assessment and programming considerations for community-dwelling older adults and those living in long-term care settings.

## Conclusion

The self-report of seasonal changes in mood and behavior and of poor illumination indoors seem to be relevant indicators of the HRQoL and mental well-being.

## Abbreviations

BDI: Beck Depression Inventory; 15D: Fifteen Dimensions Health-Related Quality of Life Instrument; GHQ: General Health Questionnaire; GSS: General Seasonality Score; HRQoL: Health Related Quality of Life; QoL: Quality of Life; SPAQ: Seasonal Pattern Assessment Questionnaire.

## Competing interests

The authors declare that they have no competing interests.

## Authors' contributions

SG initiated and drafted the manuscript together with TP. AA provided the epidemiological data collection and SS provided statistical and draft advice. JL is the principal investigator and supervisor of the manuscript. All authors critiqued revisions of the paper and approved the final manuscript. TP, SS and JL supervised SG.
